# Work-Related Musculoskeletal Disorder Prevalence by Body Area Among Nurses in Europe: Systematic Review and Meta-Analysis

**DOI:** 10.3390/jfmk10010066

**Published:** 2025-02-13

**Authors:** Philippe Gorce, Julien Jacquier-Bret

**Affiliations:** 1 Université de Toulon, CS 60584, Cedex 9, 83041 Toulon, France; gorce@univ-tln.fr; 2 International Institute of Biomechanics and Occupational Ergonomics, Avenue du Docteur Marcel Armanet, CS 10121, 83418 Hyères Cedex, France

**Keywords:** musculoskeletal disorders, body area, nurse, occupational health, safety, systematic review, meta-analysis

## Abstract

**Background/Objectives**: Nurses are highly exposed to work-related musculoskeletal disorders (WMSDs). Global prevalence exceeds 80%, and several body areas are equally at risk. Numerous studies have assessed the exposure in many countries, but few have provided an overview by continent. The aim of this study was to propose a literature review and meta-analysis to investigate the overall and body area prevalence of WMSDs among nurses in Europe. **Methods**: A systematic review and a meta-analysis were conducted between September and November 2024. Five databases were scanned without a date limit: ScienceDirect, PubMed/Medline, Google Scholar, Science.gov, and Mendeley. The article selection, review, critical appraisal, and data extraction were performed by two authors independently. Preferred Reporting Items for Systematic Reviews and Meta-Analyses (PRISMA) was used for reporting the search results. **Results**: Among the 15,751 unique identified records, 12 studies met the inclusion criteria and were included for data extraction. The studies reported a total of 5153 nurses in Europe. High heterogeneity (Cochran’s Q test and I^2^ statistic) was observed between studies. The results of the meta-analysis based on the random-effects model showed an overall prevalence of 87.8% (95% CI: 83.3–92.2%). The highest prevalence was found for lower back 61.4% (95% CI: 55.1–67.7%), neck 49.9% (95% CI: 42.9–56.8%), and upper back 46.3% (95% CI: 42.4–50.2%). **Conclusions**: Nurses are highly exposed to WMSDs, with a prevalence of over 30% for three-quarters of body areas. Due to the multitude of risk factors associated with nurses’ work, the development of recommendations is a complex multifactorial issue, requiring the exploration of different strategies to reduce the prevalence of MSDs in nurses.

## 1. Introduction

One of the major occupational health challenges of the 21st century is the reduction in musculoskeletal disorders (MSDs) [1]. MSDs are defined “as a wide range of inflammatory and degenerative conditions affecting the muscles, tendons, ligaments, joints, peripheral nerves, and supporting blood vessels. These include clinical syndromes such as tendon inflammations and related conditions (tenosynovitis, epicondylitis, bursitis), nerve compression disorders (carpal tunnel syndrome, sciatica), and osteoarthrosis, as well as less well standardized conditions such as myalgia, low back pain and other regional pain syndromes not attributable to known pathology” according to Punnett and Wegman (2004) [2]. These are present in a wide range of jobs in the primary [3], industrial [4], and tertiary [5] sectors. When these alterations are caused or aggravated by a professional activity, they are called work-related musculoskeletal disorders (WMSDs). They are responsible for a large number of direct and indirect work-related costs, as well as work stoppages [1].

Numerous studies have shown that healthcare professionals are highly exposed to WMSDs [6,7]. Among healthcare professionals, those in direct contact with patients are particularly exposed, such as physiotherapists [6], nurses [8], and nursing assistants [9], dentists [10], surgeons [11], occupational therapists [12], and midwives [13]. The overall reported prevalence is over 50% suggesting that healthcare professions are high-risk activities due to the heavy workload and the repetitive tasks performed over long periods of time. The prevalence of WMSDs among nurses worldwide is between 45% and 95%. This high prevalence can be explained by several factors linked to their occupational activity, especially the handling of patients often in awkward postures [14].

Many studies have been carried out to assess the global and body area-specific prevalence of WMSDs among nurses worldwide. They can be found in recent systemic reviews and meta-analyses conducted worldwide or in specific countries or continents. For example, Tavakkol et al. [15] focused on operating room healthcare professionals, while Clari et al. [16] proposed a synthesis for preoperative nurses. Other authors have investigated WMSD prevalence for a specific country (e.g., Saberipour et al. [17] for Iran and Wang et al. [18] for China) or continent (Kgakge et al. [19] for Africa). These synthesis studies enable prevalence to be specifically qualified according to specialty, region of the world, or other parameters. More global studies, such as Sun et al. [8], integrate all specialties to provide an overview of WMSD prevalence. However, this highly interesting study includes over 50% of cross-sectional studies carried out in China. To our knowledge, except for reports on working life [1,20], no meta-analysis has been carried out on the overall prevalence and different anatomical sites of WMSDs among all nurses in Europe.

We therefore aimed to perform a quantitative synthesis of the overall WMSD prevalence and for all body areas among nurses in Europe whatever their department or specialty. This study was conducted using a systematic review and meta-analysis to provide a reliable evidence base for the development of a health policy and the formulation of specific interventions to reduce the WMSD risk.

## 2. Materials and Methods

### 2.1. Search Strategy

We conducted a comprehensive review of the best available data using the systematic review methodology to provide an overview of the current state of MSD prevalence data among nurses. The systematic search was conducted following the Preferred Reporting Items for Systematic reviews and Meta-Analyses (PRISMA) guidelines [21] in five free databases focused on our field of study: ScienceDirect, PubMed/Medline, Google Scholar, Science.gov, and Mendeley from 12 September 2024 to 8 November 2024. The inclusion criteria were cross-sectional studies that evaluated the overall WMSD prevalence and by body area among nurses whatever their department or specialty. The keywords used for the search were the following: “work-related musculoskeletal disorders”, “nurse”, and “prevalence”. The search was entered in the databases as follows: “work-related musculoskeletal disorders” AND nurse AND prevalence. The protocol for this review was registered at PROSPERO (CRD42024625779).

The articles obtained from each database were compiled in a single file. After removing duplicates, two reviewers (P.G. and J.J.-B.) evaluated together to select the original articles. They separately screened the title/abstract/full text and assessed content according to the inclusion/exclusion criteria for final selection. Studies were excluded (1) if they were not published in English, (2) if it was not a peer-reviewed cross-sectional study, (3) if the study was not European, (4) if the results did not address the prevalence of MSDs in nurses, (5) if the results were not sufficiently detailed by body area, and (6) if the number of assessed body areas was less than 3. All discrepancies were resolved by consensus and re-review of the articles.

### 2.2. Quality Assessment Data Extraction

The Appraisal tool for Cross-Sectional Studies (AXIS tool) [22] was used to perform the quality appraisal of the included articles. Each of the criteria was evaluated on its presence (“Yes”) or absence (“No”) to compute the percentage of items present. The classification proposed by Hermanson and Choi [23] was applied to qualify each article as follows: 0–50% of items present correspond to a high risk of bias; 50–80% correspond to a medium risk of bias; and 80–100% to a low risk of bias. Two reviewers (P.G. and J.J.-B.) performed the quality assessment separately. The data were reported in a summary table by each reviewer and summarized in a final table. The discrepancies have been discussed for the final evaluation, involving a third reviewer where necessary.

### 2.3. Data Extraction

Data extracted included number of participants, response rate, male/female distribution, average age, height, weight and body mass index (BMI) of nurses, country, number of years in practice, work sector, and WMSD prevalence by body area. The main outcomes were the overall prevalence and the prevalence for ten body areas: the neck, back, upper back, mid-back, lower back, shoulders, elbows, wrists, hips, knees, and ankles. All data were summarized in a table (white boxes indicate missing data). When a parameter was formulated on the basis of a subgroup from the main sample, it was recalculated in relation to the total sample so that it could be compared with those from other studies.

### 2.4. Statistical Analysis

The meta-analysis was performed based on the work of Neyeloff et al. [24]. The heterogeneity of the studies was assessed using Cochran’s Q test (significance level < 10%) and I^2^ statistic (significance level > 50%). In case of heterogeneity, random effects model with inverse-variance approach was employed. Otherwise, the fixed effects model was applied.

## 3. Results

### 3.1. Search Results

**Figure 1:**
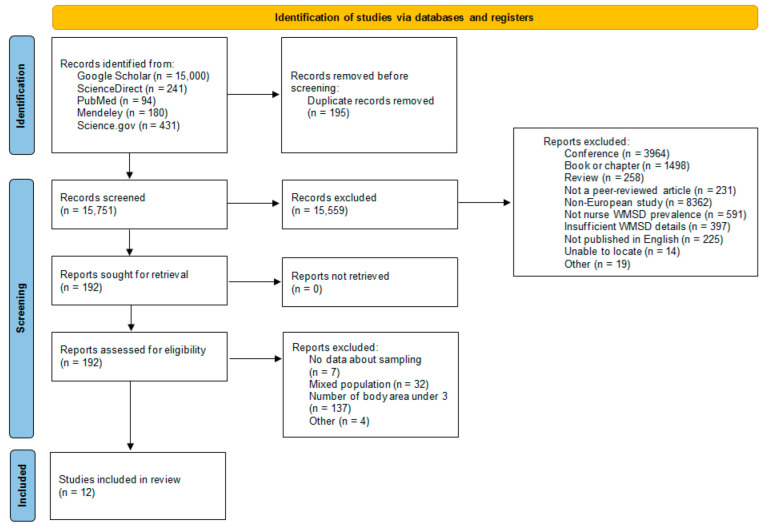
PRISMA flow diagram.

The research in the five databases identified 15,946 records. After removing duplicates, 15,751 unique articles were identified and 15,559 were excluded were excluded from the selection due to their format (conference, review, book…), their non-European origin, and the fact that they did not explicitly address the prevalence of WMSDs in nurses. Of the remaining 192 articles, 180 were excluded after full reading, either because sample information was not available, or because the sample was composed of mixed populations without being able to differentiate between them, or because the number of body areas was less than 3. Twelve cross-sectional studies with a total of 5153 nurses were finally included in the present review. The search process is presented in Figure 1.

### 3.2. Quality Appraisal

The AXIS tool was used to perform the quality assessment. Of the 12 articles included, 2 had a low risk of bias, i.e., Koyuncu et al. [25] and Sezgin et al. [26] (more than 80% of items present). The others had a medium risk of bias (between 50 and 80% of items present) [27,28,29,30,31,32,33,34,35]. Details of the analysis are presented in Table 1.

### 3.3. Study Characteristics

Table 2 presents data from the 12 included studies conducted in seven different European countries: Denmark, Estonia, Greece, Portugal, Sweden, Switzerland, and Turkey. All were cross-sectional studies of the overall risk of WMSDs in nurses, and by body area. Sample sizes were highly heterogeneous, ranging from 105 [25] to 1396 [34] participants. Response rates also varied widely from one study to another, ranging from 3.8% [32] to 84.0% [27,28]. Nine of the studies were conducted with a mixed population with a large majority of women. One study included only women [29] and two studies did not provide information on the sample composition [30,31]. The mean age of participants ranged from 27.9 ± 5.1 [26] to 47.6 ± 11.1 [35] years, with the year of practice ranging from 6.4 ± 4.9 to 20.6 ± 11.1 in the same two studies. Only half the studies [25,26,30,34,35] reported an average weekly activity between 32.9 ± 3.9 and 49.2 ± 10.5 h/week. Few studies provided information on morphological characteristics: three reported average height [27,28,34], only one reported average weight [34], and five reported average body mass index [25,27,28,29,32].

**Table 1:** Quality appraisal of the included cross-sectional studies according to the AXIS tool.

	1. Were the aims/objectives of the study clear?	2. Was the study design appropriate for the stated aim(s)?	3. Was the sample size justified?	4. Was the target/reference population clearly defined? (Is it clear who the research was about?)	5. Was the sample frame taken from an appropriate population base so that it closely represented the target/reference population under investigation?	6. Was the selection process likely to select subjects/participants that were representative of the target/reference population under investigation?	7. Were measures undertaken to address and categorise non-responders?	8. Were the risk factor and outcome variables measured appropriate to the aims of the study?	9. Were the risk factor and outcome variables measured correctly using instruments/measurements that had been trialled, piloted or published previously?	10. Is it clear what was used to determined statistical significance and/or precision estimates? (eg, p values, CIs)	11. Were the methods (including statistical methods) sufficiently described to enable them to be repeated?	12. Were the basic data adequately described?	13. Does the response rate raise concerns about non-response bias?	14. If appropriate, was information about non-responders described?	15. Were the results internally consistent?	16. Were the results for the analyses described in the methods, presented?	17. Were the authors’ discussions and conclusions justified by the results?	18. Were the limitations of the study discussed?	19. Were there any funding sources or conflicts of interest that may affect the authors’ interpretation of the results?	20. Was ethical approval or consent of participants attained?	Yes	No	Yes (%)	Risk of biais
Alexopoulos et al., 2003 [27]	Yes	Yes	No	Yes	Yes	Yes	No	Yes	Yes	Yes	Yes	Yes	No	NA	Yes	Yes	Yes	No	No	Yes	14	5	74%	Medium
Alexopoulos et al., 2006 [28]	Yes	Yes	No	Yes	Yes	Yes	No	Yes	Yes	Yes	Yes	Yes	No	NA	Yes	Yes	Yes	No	No	Yes	14	5	74%	Medium
Arvidsson et al., 2016 [29]	Yes	Yes	No	Yes	Yes	Yes	No	Yes	Yes	Yes	Yes	Yes	No	NA	Yes	Yes	Yes	No	No	Yes	14	5	74%	Medium
Freimann et al., 2013 [30]	Yes	Yes	No	Yes	Yes	Yes	No	Yes	Yes	Yes	Yes	Yes	No	NA	Yes	Yes	Yes	Yes	No	Yes	15	4	79%	Medium
Koyuncu et al., 2024 [25]	Yes	Yes	Yes	Yes	Yes	Yes	No	Yes	Yes	Yes	Yes	Yes	No	NA	Yes	Yes	Yes	Yes	No	Yes	16	3	84%	Low
Nützi et al., 2015 [31]	Yes	Yes	No	Yes	Yes	Yes	No	Yes	Yes	Yes	Yes	Yes	No	NA	Yes	Yes	Yes	Yes	No	Yes	15	4	79%	Medium
Passali et al., 2018 [32]	Yes	Yes	No	Yes	Yes	Yes	No	Yes	Yes	Yes	Yes	Yes	No	NA	Yes	Yes	Yes	Yes	No	Yes	15	4	79%	Medium
Ribeiro et al., 2017 [33]	Yes	Yes	No	Yes	Yes	Yes	No	Yes	Yes	Yes	Yes	Yes	No	NA	Yes	Yes	Yes	Yes	No	Yes	15	4	79%	Medium
Serranheira et al., 2015 [34]	Yes	Yes	No	Yes	Yes	Yes	No	Yes	Yes	Yes	Yes	Yes	No	NA	Yes	Yes	Yes	Yes	No	Yes	15	4	79%	Medium
Sezgin et al., 2015 [26]	Yes	Yes	Yes	Yes	Yes	Yes	No	Yes	Yes	Yes	Yes	Yes	No	NA	Yes	Yes	Yes	Yes	No	Yes	16	3	84%	Low
Westergren et al., 2021 [35]	Yes	Yes	No	Yes	Yes	Yes	No	Yes	Yes	Yes	Yes	Yes	No	NA	Yes	Yes	Yes	Yes	No	Yes	15	4	79%	Medium
Westergren et al., 2021 [35]	Yes	Yes	No	Yes	Yes	Yes	No	Yes	Yes	Yes	Yes	Yes	No	NA	Yes	Yes	Yes	Yes	No	Yes	15	4	79%	Medium

### 3.4. Meta-Analysis Results—Annual Prevalence of WMSDs Among Nurses

**Figure 2:**
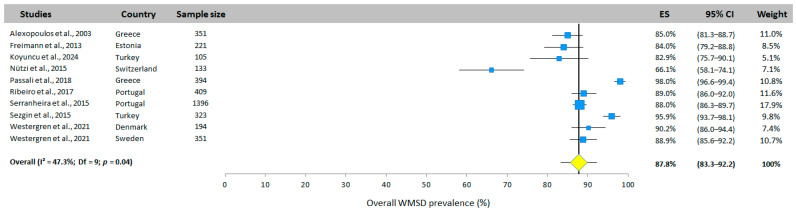
Overall WMSD prevalence. Each study is represented by a square. The horizontal line represents 95% CI. The diamond represents the total prevalence according to the random effects model [25,26,27,30,31,32,33,34,35].

The overall WMSD prevalence among nurses was presented in 10 of the 12 included studies, covering 8 different countries (Figure 2). Based on the random-effects model, the overall prevalence was 87.8% (95% CI: 83.3–92.2%). All studies had an overall prevalence of over 80%, with the exception of Nutzi et al. [31], which reported a prevalence of 66.1%.

### 3.5. Meta-Analysis Results—Annual Prevalence of WMSDs by Body Area

The top three locations for WMSDs among nurses were the lower back (61.4%), neck (49.9%), and upper back (in 46.3%). Shoulder and wrist prevalence ranged from 30 to 40%, while the lower limb prevalence was between 20 and 30%, with knee prevalence being the highest. The elbow ranked last, with a prevalence of less than 15%. The included studies showed high heterogeneity. The following sections detail the results for each body area.

#### 3.5.1. Neck WMSD Prevalence

**Figure 3:**
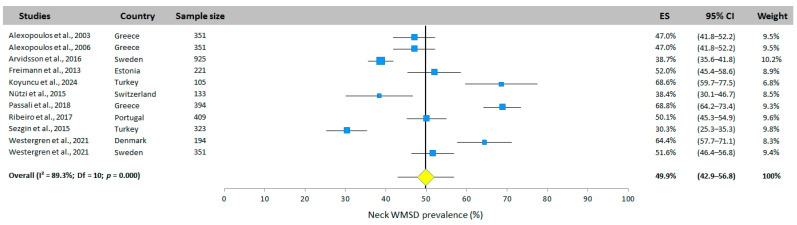
Neck WMSD prevalence. Each study is represented by a square. The horizontal line represents 95% CI. The diamond represents the total prevalence according to the random effects model [25,26,27,28,29,30,31,32,33,35].

The neck WMSD prevalence was mentioned in 11 of the 12 included studies, covering eight different countries (Figure 3). The results were heterogeneous, with prevalence ranging from 30.3% [26] to 68.8% [32]. The overall prevalence was 49.9% (95% CI: 42.9–56.8%, random-effects model).

#### 3.5.2. Upper-Back WMSD Prevalence

**Figure 4:**
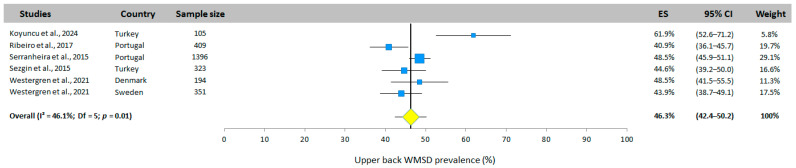
Upper-back WMSD prevalence. Each study is represented by a square. The horizontal line represents 95% CI. The diamond represents the total prevalence according to the random effects model [25,26,33,34,35].

The prevalence of upper-back WMSD is presented in Figure 4. Only half the studies investigated this body area. The total prevalence obtained from the random effects model was 46.3% (95% CI: 42.4–50.2%). Only the study by Koyuncu et al. [25] reported a higher prevalence than the others (61.9%).

#### 3.5.3. Lower-Back WMSD Prevalence

**Figure 5:**
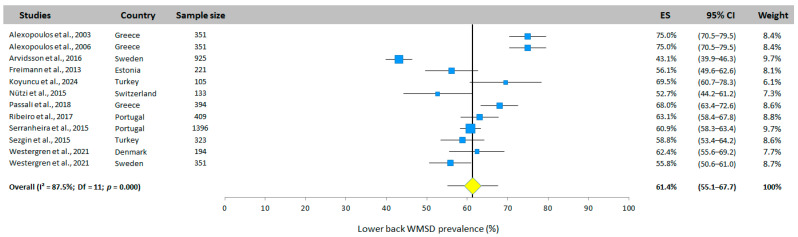
Lower-back WMSD prevalence. Each study is represented by a square. The horizontal line represents 95% CI. The diamond represents the total prevalence according to the random effects model [25,26,27,28,29,30,31,32,33,34,35].

The total prevalence of lower-back MSD was the highest at 61.4% (95% CI: 55.1–67.7%, random effects model). All the studies included in the analysis (12 studies from 8 different countries) reported a prevalence for this area with values ranging from 43.1% to 75.0%. Figure 5 depicts the detailed results.

#### 3.5.4. Shoulder WMSD Prevalence

**Figure 6:**
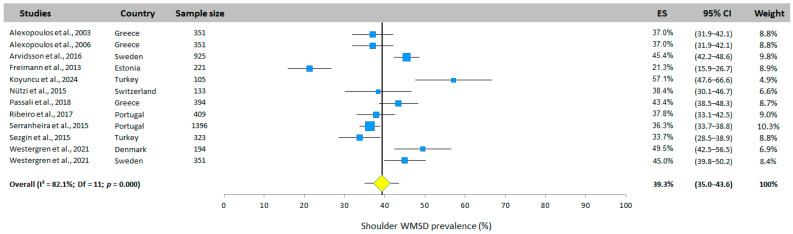
Shoulder WMSD prevalence. Each study is represented by a square. The horizontal line represents 95% CI. The diamond represents the total prevalence according to the random effects model [25,26,27,28,29,30,31,32,33,34,35].

The shoulder WMSD prevalence was investigated by all 12 studies (Figure 6). The overall prevalence was 39.3% (95% CI: 35.0–43.6%, random-effects model). The studies by Freimann et al. [30] and Koyuncu et al. [25] showed results far from this mean value, with prevalences of 21.3% and 57.1%, respectively.

#### 3.5.5. Elbow WMSD Prevalence

**Figure 7:**
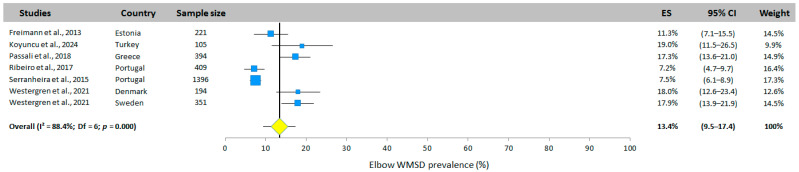
Elbow WMSD prevalence. Each study is represented by a square. The horizontal line represents 95% CI. The diamond represents the total prevalence according to the random effects model [25,30,32,33,34,35].

Seven studies (in six different countries) presented a prevalence of MSD for the elbow. The mean prevalence obtained using the random effects model was 13.4% (95% CI: 9.5–17.4%, Figure 7), with values ranging from 7.2% to 19.0%.

#### 3.5.6. Wrist WMSD Prevalence

**Figure 8:**
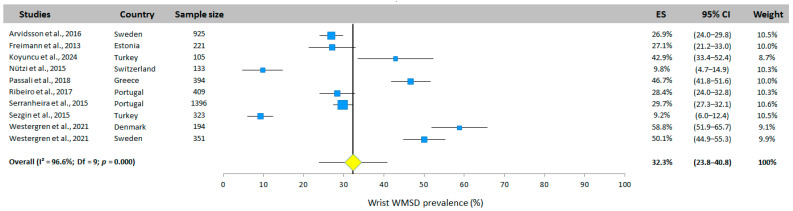
Wrist WMSD prevalence. Each study is represented by a square. The horizontal line represents 95% CI. The diamond represents the total prevalence according to the random effects model [25,26,29,30,31,32,33,34,35].

The prevalence of WMSD at the wrist was studied in 10 studies (7 different countries, Figure 8). The overall prevalence was 32.3% (95% CI: 23.8–40.8%, random effects model). The results were highly heterogeneous. The studies by Nutzi et al. [31] and Sezgin et al. [26] reported a low prevalence (9.8% and 9.2%, respectively), while Westergren et al. [35] observed a significantly higher prevalence in Sweden (50.1%) and Denmark (58.8%).

#### 3.5.7. Hip WMSD Prevalence

**Figure 9:**
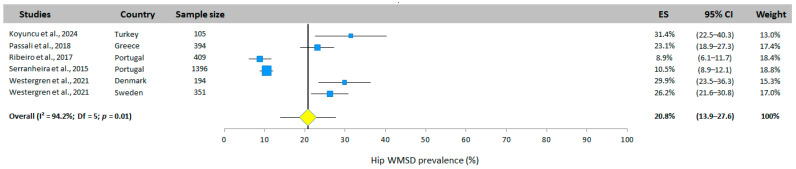
Hip WMSD prevalence. Each study is represented by a square. The horizontal line represents 95% CI. The diamond represents the total prevalence according to the random effects model [25,32,33,34,35].

The overall prevalence of WMSD for the hip obtained from six studies (five different countries) was 20.8% (95% CI: 13.9–27.6%, random effects model, Figure 9) with values between 8.9% [33] and 31.4% [25].

#### 3.5.8. Knee WMSD Prevalence

**Figure 10:**
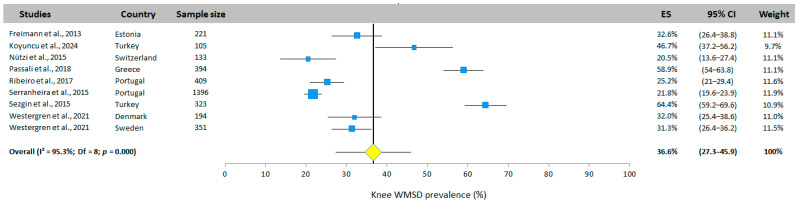
Knee WMSD prevalence. Each study is represented by a square. The horizontal line represents 95% CI. The diamond represents the total prevalence according to the random effects model [25,26,30,31,32,33,34,35].

Nine studies (seven different countries) reported the prevalence of knee WMSD. The overall prevalence was estimated at 36.6% (95% CI: 27.3–45.9%, random effects model, Figure 10). Significant heterogeneity was observed between the studies, with values ranging from 20.5% [31] to 64.4% [26].

#### 3.5.9. Ankle WMSD Prevalence

**Figure 11:**
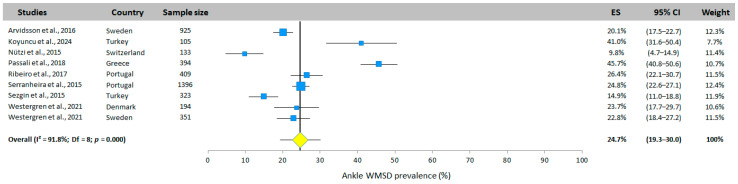
Ankle WMSD prevalence. Each study is represented by a square. The horizontal line represents 95% CI. The diamond represents the total prevalence according to the random effects model [25,26,29,31,32,33,34,35].

Ankle prevalence was investigated in nine studies (six countries). The total prevalence was 27.4% (95% CI: 19.3–30.0%, random effects model, Figure 11). The studies by Nutzi et al. [31] and Passali et al. [32] presented the two extreme values, with a prevalence of 9.8% and 45.7%, respectively.

## 4. Discussion

The aim of this study was to propose a literature review and meta-analysis to investigate the prevalence of WMSD among nurses in Europe. The objective was to analyze the overall WMSD prevalence and the prevalence by body area. Twelve studies were included in the analysis.

The overall WMSD prevalence assessed by the random effects model had a high value of 87.8% (95% CI: 83.3–92.2%). In the literature, the only work relating to one continent is that of Kgakge et al. for the sub-Saharan Africa [19]. The authors reported that, among the 29 included studies, 18 of them reported evidence about the 12-month prevalence of WMSD. As the aim of this work was a scoping review, the overall prevalence was presented as a range from 57.1% up to 95.7%. At present, there is no synthesis of global prevalence by continent for nurses. Conversely, more data are available in the international literature concerning the different body areas.

The meta-analysis showed that the highest European prevalence was for lower back with 61.4% (95% CI: 55.1–67.7%), neck with 49.9% (95% CI: 42.9–56.8%), and upper back with 46.3% (95% CI: 42.4–50.2%). In their scoping review, Kgakge et al. [19] only reported the prevalence for lower back in sub-Saharan Africa, with a range between 32.5% (Kenya [36]) and 87.5% (Sudan [37]). To our knowledge, there is no synthesis reporting the prevalence of WMSDs by body area among nurses on other continents. However, surveys have been conducted in many countries around the world. Among the most well-documented studies are the meta-analyses carried out in Asia, particularly those by Wang et al. in China (23 articles, 21,042 nurses [18]) and Saberipour et al. in Iran (33 articles, 11,995 nurses [17]). Saberipour et al. only determined the overall prevalence of the lower back with a value of 60% (95% CI: 60–61%), which is very close to that of the present study [17]. Wang et al. reported prevalence for eight body zones [18]. For the elbow and the three areas of the lower limb, the prevalence are very close to those of the present study in Europe: 15% for the elbow (95% CI: 11–20%), 21% for the hip (95% CI: 18–25%), 31% for the knee (95% CI: 26–37%), and 30% for the ankle (95% CI: 25–34%) [18]. However, for the neck and shoulder, the values of the present study were lower than those of Wang et al. (49.9% vs. 58% and 39.3% vs. 49%, respectively), while for the wrist, our results were slightly higher (32.3% vs. 25%) [18].

In a broader context, it is interesting to compare the results of the present study with those presented in the literature worldwide. Two studies carried out a systematic review with a meta-analysis of the prevalence of nine body areas in nurses, including studies from different continents. Clari et al. focused on perioperative nurses [16], while Sun et al. considered all nurses, but 50% of the included studies were Chinese [8]. Firstly, all authors agree that the most prevalent body area is the lower back, with a value of 60%. Similarly, authors agree that the elbow is the least affected area, with a similar prevalence of 18% for Clari et al. [16] and Sun et al. [8], the prevalence reported in this study is less (13.4%). For the next three areas, our results are close to those reported by Sun et al., with a prevalence of between 40% and 50% for the neck, shoulder, and upper back [8]. In contrast, Clari et al. present a different classification, with knee and hip ranking ahead of neck and upper back with similar prevalence [16].

In more detail, the results of our studies showed slightly higher values for the upper back (46.3% (95% CI: 42.4–50.2%)) and wrist (32.3% (95% CI: 23.8–40.8%)) than those reported by Clari (34% (95% CI: 25–44%) and 29% (95% CI: 20–40%), respectively) and Sun et al. (43.3% (95% CI: 38.6–48.1%) and 30.8% (95% CI: 25.4–36.3%), respectively) [8]. Three prevalences, i.e., those for neck (49.9% (95% CI: 42.9–56.8%)), lower back (61.4% (95% CI: 55.1–67.7%)), and knee (36.6% (95% CI: 27.3–45. 9%)), were between the values proposed by the two authors (Clari et al.: 39% (CI 95%: 29–51%), 62% (CI 95%: 54–70%), and 47% (CI 95%: 36–59%) [16]; Sun et al.: 53.0% (CI 95%: 45.8–60.3%), 59.5% (CI 95%: 53.6–65.4%) and 35.9% (CI 95%: 29.5–42.3%), respectively [8]). Finally, for shoulder (39.3% (95% CI: 35.0–43.6%)), elbow (13.4% (95% CI: 9.5–17.4%)), hip (20.8% (95% CI: 13.9–27.6%)), and ankle (27.4% (95% CI: 19.3–30.0%)), our results were lower (Clari et al.: 44% (95% CI: 37–51%), 18% (95% CI: 12–26%), 42% (95% CI: 31–53%), 35% (95% CI: 22–51%) [16]; Sun et al.: 46.8% (95% CI: 40.3–53.3%), 18.3% (95% CI: 13.6–22.9%), 28.9% (95% CI: 22.9–34.8%), and 33.0% (95% CI: 26.4–39.6%), respectively [8]). Finally, with regard to overall prevalence, the value presented in this study is 10% higher than that reported by Sun et al. (87.8% (95% CI: 83.3–92.2%) vs. 77.2% (95% CI: 72.5–81.9%) [8]), as the study by Clari et al. did not report overall prevalence [16].

This high prevalence of WMSDs among nurses is linked to their daily activities. Numerous risk factors have been reported, such as high psychosocial demands including low job control [38], manual handling of patients (patient lifting and transfer), and awkward postures repeated several times a day over long working hours. This prevalence is similar to that observed in other healthcare professionals, despite notable differences in daily work activities. A recent study compared the prevalence of WMSDs among surgeons in America, Asia, and Europe [39].

European surgeons showed a higher prevalence than European nurses for the neck (54.1% vs. 49.9%) and shoulder (51.4% vs. 39.3%). This may be explained by the specificity of these operations, which require a very high level of concentration, precision, and high workloads [40,41,42]. As a result, surgeons repeat and maintain awkward postures for long periods of time, particularly with significant neck flexion and shoulder strain. Back (58.7% vs. 46.3% and 61.4% for upper back and lower back, respectively for nurses) and wrist (31.8% vs. 32.3%) prevalence is equivalent between the two professions. On the other hand, knee prevalence is much higher among nurses (36.6% vs. 26.7%), largely due to the many displacements they make during their long working time.

Exposure to WMSDs is becoming more widespread worldwide. As with nurses, back, neck, and shoulder areas are widely exposed among surgeons [11,43], but also among other healthcare professionals such as physiotherapists [44,45], dentists [46,47], or midwives [13,48] with a prevalence of over 40%.

Regarding the results reported in the various studies, significant heterogeneity was observed (I^2^ > 80%). This result underlines that this parameter is essential to manage in meta-analyses. Indeed, the sample size and the sensitivity of the tools used (especially the type of questionnaire) are parameters that could explain this heterogeneity. In addition, the diversity of nurses’ profiles (age, gender, experience…), their geographical location (country and continent), and their working conditions (department, workload, working hours…) are all factors that can affect the prevalence of WMSDs. Despite this heterogeneity, our analysis showed that 9 out of 10 nurses in Europe are likely to report an MSD in at least one body area, and almost one in two is likely to report it in the back, neck, or shoulders.

Some limitations could be addressed. The first limitation concerns sample size. On the one hand, studies investigating the prevalence of WMSDs in nurses by body area in Europe are few in number. This number could perhaps have been higher if other databases such as Scopus or Web of Science had been included. On the other, there is considerable variability in the sample (105 to 1396). Although meta-analyses have weighted the results, it would be advisable to reduce this difference and increase the number of studies to assess the prevalence of WMSDs.

The second limitation concerns the method of data collection. Although the majority of studies used the Nordic Musculoskeletal Disorders Questionnaire [49], others used a modified version or a different questionnaire. Differences in wording may therefore lead to variations in the understanding and interpretation of the questions, and consequently to variations in the assessment of the WMSD prevalence. A sub-group analysis could have compensated for this diversity, but the number of studies available does not allow it.

The third limitation relates to the search strategy. A minimum limit of three body areas was chosen in order to conduct a meta-analysis on the largest number of body areas. This criterion excluded all studies that focused on just one area, the most represented being the lower back. The addition of other criteria such as the limitation to articles written in English or to the “original article” could have led to the exclusion or potential omission of works that could have completed and extended the results of the present review and meta-analysis.

The present study has provided an overview of work carried out on the WMSD prevalences among European nurses, both overall and by body zone. The results clearly show that more still needs to be done to reduce exposure to WMSDs, particularly in the lower back, upper back, neck, and shoulders. Nurses therefore remain a population at risk, and we must continue to sensitize and educate them. Their environment must also be adapted, and new technological assistance solutions developed. These are the key areas for future research.

## 5. Conclusions

European nurses are significantly exposed to WMSDs (87.8%). The meta-analyses showed that the most affected areas were the neck, upper and lower back, and shoulders with prevalences between 40 and 60%. There seems to be a consensus that the lower back is the most exposed area, with a prevalence of around 60%. Similarly, the elbow is the least exposed, with a prevalence of 18%. Due to the multitude of risk factors associated with nurses’ work, the development of recommendations is a complex multifactorial issue, requiring the exploration of different strategies to reduce the prevalence of WMSDs in nurses. Future work could focus on work conditions, especially on the various tasks performed by nurses during their working time, and on postural analysis to reduce the appearance of WMSDs. Subgroup analyses could reduce the variability observed and improve the results, assuming a sufficient number of studies.

## Figures and Tables

**Table 2:** Characteristics of the 12 included cross-sectional studies about WMSDs among nurses. Overall WMSD prevalence and prevalence by body area was reported for each study (when available).

Authors	Study Characteristics				Body Area WMSD Prevalence									WMSD Overall Prevalence
					Neck	Upper Back	Lower Back	Shoulder	Elbow	Wrist/Hand	Hip/Thigh	Knee	Ankle/Foot	
**Alexopoulos et al., 2003** [27]	**Population**	Nurse	**Experience (year)**	12.7 ± 6.4	47.0%		75.0%	37.0%						85.0%
	**N-participant**	351	**Sector**	Gov										
	**Response rate**	84%	**Practice (h/week)**	-										
	**Male/female**	19.1%/80.9%	**Height (m)**	166.4 ± 6.9										
	**Age (year, mean ± SD)**	37.07 ± 7.3	**Weight (kg)**	-										
	**Country**	Greece	**BMI**	24.3 ± 3.6										
**Alexopoulos et al., 2006** [28]	**Population**	Nurse	**Experience (year)**	12.7 ± 7	47.0%		75.0%	37.0%						
	**N-participant**	351	**Sector**	Gov										
	**Response rate**	84%	**Practice (h/week)**	-										
	**Male/female**	19%/81.0%	**Height (m)**	166.4 ± 8.1										
	**Age (year, mean ± SD)**	37.1 ± 7.3	**Weight (kg)**	-										
	**Country**	Greece	**BMI**	24.3 ± 4.1										
**Arvidsson et al., 2016** [29]	**Population**	Nurse	**Experience (year)**	-	38.7%		43.1%	45.4%		26.9%			20.1%	
	**N-participant**	925	**Sector**	-										
	**Response rate**	77%	**Practice (h/week)**	-										
	**Male/female**	0.0%/100%	**Height (m)**	-										
	**Age (year, mean ± SD)**	-	**Weight (kg)**	-										
	**Country**	Sweden	**BMI**	24.35										
**Freimann et al., 2013** [30]	**Population**	Nurse	**Experience (year)**	-	52.0%		56.1%	21.3%	11.3%	27.1%		32.6%		84.0%
	**N-participant**	221	**Sector**	Gov										
	**Response rate**	57.00%	**Practice (h/week)**	40.5 ± 6.7										
	**Male/female**	-	**Height (m)**	-										
	**Age (year, mean ± SD)**	38.7 ± 10.2	**Weight (kg)**	-										
	**Country**	Estonia	**BMI**	-										
**Koyuncu et al., 2024** [25]	**Population**	Nurse	**Experience (year)**	14.11 ± 9.40	68.6%	61.9%	69.5%	57.1%	19.0%	42.9%	31.4%	46.7%	41.0%	82.9%
	**N-participant**	105	**Sector**	Gov										
	**Response rate**	-	**Practice (h/week)**	49.15 ± 10.48										
	**Male/female**	24.8%/75.2%	**Height (m)**	-										
	**Age (year, mean ± SD)**	36.55 ± 8.51	**Weight (kg)**	-										
	**Country**	Turkey	**BMI**	25.35 ± 3.69										
**Nützi et al., 2015** [31]	**Population**	Nurse	**Experience (year)**	15 ± 10.6	38.4%		52.7%	38.4%		9.8%		20.5%	9.8%	66.1%
	**N-participant**	133	**Sector**	Gov										
	**Response rate**	43%	**Practice (h/week)**	-										
	**Male/female**	-	**Height (m)**	-										
	**Age (year, mean ± SD)**	39.94 ± 11.9	**Weight (kg)**	-										
	**Country**	Switzerland	**BMI**	-										
**Passali et al., 2018** [32]	**Population**	Nurse	**Experience (year)**	13.07 ± 7.88	68.8%		68.0%	43.4%	17.3%	46.7%	23.1%	58.9%	45.7%	98.0%
	**N-participant**	394	**Sector**	Gov										
	**Response rate**	3.82%	**Practice (h/week)**	-										
	**Male/female**	19.4%/80.6%	**Height (m)**	-										
	**Age (year, mean ± SD)**	37.85 ± 7.48	**Weight (kg)**	-										
	**Country**	Greece	**BMI**	24.49 ± 4.48										
**Ribeiro et al., 2017** [33]	**Population**	Nurse	**Experience (year)**	15.6	50.1%	40.9%	63.1%	37.8%	7.2%	28.4%	8.9%	25.2%	26.4%	89.0%
	**N-participant**	409	**Sector**	-										
	**Response rate**	5.4%	**Practice (h/week)**	-										
	**Male/female**	16%/84%	**Height (m)**	-										
	**Age (year, mean ± SD)**	39.5 ± 8.8	**Weight (kg)**	-										
	**Country**	Portugal	**BMI**	-										
**Serranheira et al., 2015** [34]	**Population**	Nurse	**Experience (year)**	13.25 ± 8.9		48.5%	60.9%	36.3%	7.5%	29.7%	10.5%	21.8%	24.8%	88.0%
	**N-participant**	1396	**Sector**	-										
	**Response rate**	-	**Practice (h/week)**	41.14 ± 8.9										
	**Male/female**	24.2%/75.8%	**Height (m)**	165.4 ± 11.04										
	**Age (year, mean ± SD)**	37.2 ± 9.16	**Weight (kg)**	67.02 ± 13.46										
	**Country**	Portugal	**BMI**	-										
**Sezgin et al., 2015** [26]	**Population**	Nurse	**Experience (year)**	6.4 ± 4.9	30.3%	44.6%	58.8%	33.7%		9.2%		64.4%	14.9%	95.9%
	**N-participant**	323	**Sector**	Gov/Private										
	**Response rate**	-	**Practice (h/week)**	47.2 ± 8.9										
	**Male/female**	20.7%/79.3%	**Height (m)**	-										
	**Age (year, mean ± SD)**	27.9 ± 5.1	**Weight (kg)**	-										
	**Country**	Turkey	**BMI**	-										
**Westergren et al., 2021** [35]	**Population**	Nurse	**Experience (year)**	20.6 ± 11.1	64.4%	48.5%	62.4%	49.5%	18.0%	58.8%	29.9%	32.0%	23.7%	90.2%
	**N-participant**	194	**Sector**	-										
	**Response rate**	40.20%	**Practice (h/week)**	32.9 ± 3.8										
	**Male/female**	2.6%/97.4%	**Height (m)**	-										
	**Age (year, mean ± SD)**	47.6 ± 11.1	**Weight (kg)**	-										
	**Country**	Denmark	**BMI**	-										
**Westergren et al., 2021** [35]	**Population**	Nurse	**Experience (year)**	18.9 ± 10.6	51.6%	43.9%	55.8%	45.0%	17.9%	50.1%	26.2%	31.3%	22.8%	88.9%
	**N-participant**	351	**Sector**	-										
	**Response rate**	64.90%	**Practice (h/week)**	35.6 ± 3.9										
	**Male/female**	7.8%/92.2%	**Height (m)**	-										
	**Age (year, mean ± SD)**	45.4 ± 10.5	**Weight (kg)**	-										
	**Country**	Sweden	**BMI**	-										

Abbreviation: Gov: governmental; BMI: body mass index; WMSD: work-related musculoskeletal disorders.

## References

[1] EU-OSHA Les Troubles Musculo-Squelettiques d’Origine Professionnelle: Faits et Chiffres—Rapport de Synthèse Compilé à Partir de 10 Rapports d’États Membres de l’UE. https://osha.europa.eu/fr/publications/work-related-musculoskeletal-disorders-facts-and-figures-synthesis-report-10-eu-member/view.

[2] Punnett L., Wegman D.H. (2004). Work-related musculoskeletal disorders: The epidemiologic evidence and the debate. J. Electromyogr. Kinesiol..

[3] Akbar K.A., Try P., Viwattanakulvanid P., Kallawicha K. (2023). Work-Related Musculoskeletal Disorders Among Farmers in the Southeast Asia Region: A Systematic Review. Saf. Health Work..

[4] He X., Xiao B., Wu J., Chen C., Li W., Yan M. (2023). Prevalence of work-related musculoskeletal disorders among workers in the automobile manufacturing industry in China: A systematic review and meta-analysis. BMC Public Health.

[5] Demissie B., Bayih E.T., Demmelash A.A. (2024). A systematic review of work-related musculoskeletal disorders and risk factors among computer users. Heliyon.

[6] Jacquier-Bret J., Gorce P. (2023). Prevalence of Body Area Work-Related Musculoskeletal Disorders among Healthcare Professionals: A Systematic Review. Int. J. Environ. Res. Public Health.

[7] Suganthirababu P., Parveen A., Mohan Krishna P., Sivaram B., Kumaresan A., Srinivasan V., Vishnuram S., Alagesan J., Prathap L. (2023). Prevalence of work-related musculoskeletal disorders among health care professionals: A systematic review. Work.

[8] Sun W., Yin L., Zhang T., Zhang H., Zhang R., Cai W. (2023). Prevalence of Work-Related Musculoskeletal Disorders among Nurses: A Meta-Analysis. Iran. J. Public Health.

[9] Ching S.S.Y., Szeto G., Lai G.K.B., Lai X.B., Chan Y.T., Cheung K. (2018). Exploring the Synergic Effects of Nursing Home Work on Work-Related Musculoskeletal Disorders Among Nursing Assistants. Workplace Health Saf..

[10] Lietz J., Kozak A., Nienhaus A. (2018). Prevalence and occupational risk factors of musculoskeletal diseases and pain among dental professionals in Western countries: A systematic literature review and meta-analysis. PLoS ONE.

[11] Epstein S., Sparer E.H., Tran B.N., Ruan Q.Z., Dennerlein J.T., Singhal D., Lee B.T. (2018). Prevalence of Work-Related Musculoskeletal Disorders Among Surgeons and Interventionalists: A Systematic Review and Meta-analysis. JAMA Surg..

[12] Aydoner Bektas S., Özata Değerli M., Altuntaş O., Bumin G. (2024). Work-Related Musculoskeletal Disorders Among Occupational Therapists in Turkey: A Cross-Sectional Study. Occup. Ther. Health Care.

[13] Okuyucu K., Gyi D., Hignett S., Doshani A. (2019). Midwives are getting hurt: UK survey of the prevalence and risk factors for developing musculoskeletal symptoms. Midwifery.

[14] Bos E., Krol B., van der Star L., Groothoff J. (2007). Risk factors and musculoskeletal complaints in non-specialized nurses, IC nurses, operation room nurses, and X-ray technologists. Int. Arch. Occup. Environ. Health.

[15] Tavakkol R., Karimi A., Hassanipour S., Gharahzadeh A., Fayzi R. (2020). A Multidisciplinary Focus Review of Musculoskeletal Disorders Among Operating Room Personnel. J. Multidiscip. Healthc..

[16] Clari M., Godono A., Garzaro G., Voglino G., Gualano M.R., Migliaretti G., Gullino A., Ciocan C., Dimonte V. (2021). Prevalence of musculoskeletal disorders among perioperative nurses: A systematic review and META-analysis. BMC Musculoskelet. Disord..

[17] Saberipour B., Ghanbari S., Zarea K., Gheibizadeh M., Zahedian M. (2019). Investigating prevalence of musculoskeletal disorders among Iranian nurses: A systematic review and meta-analysis. Clin. Epidemiol. Glob. Health.

[18] Wang K., Zeng X., Li J., Guo Y., Wang Z. (2024). The prevalence and risk factors of work-related musculoskeletal disorders among nurses in China: A systematic review and meta-analysis. Int. J. Nurs. Stud..

[19] Kgakge K., Hlongwa M., Nwagbara U.I., Ginindza T. (2024). The distribution of work-related musculoskeletal disorders among nurses in sub-Saharan Africa: A scoping review. Syst. Rev..

[20] Eurofound Sixth European Working Conditions Survey—Overview Report. https://www.eurofound.europa.eu/en/publications/2016/sixth-european-working-conditions-survey-overview-report.

[21] Harris J.D., Quatman C.E., Manring M.M., Siston R.A., Flanigan D.C. (2014). How to Write a Systematic Review. Am. J. Sports Med..

[22] Downes M.J., Brennan M.L., Williams H.C., Dean R.S. (2016). Development of a critical appraisal tool to assess the quality of cross-sectional studies (AXIS). BMJ Open.

[23] Hermanson J.E., Choi S.D. (2012). Study of musculoskeletal risks of the office-based surgeries. Work.

[24] Neyeloff J.L., Fuchs S.C., Moreira L.B. (2012). Meta-analyses and Forest plots using a microsoft excel spreadsheet: Step-by-step guide focusing on descriptive data analysis. BMC Res. Notes.

[25] Koyuncu A., Kaya K., Kaya O., Yava A. (2024). The Impact of Work-Related Musculoskeletal Pains on Routine Tasks Among Operating Room Nurses: A Multicenter Cross-Sectional Study. Pain. Manag. Nurs..

[26] Sezgin D., Esin M.N. (2015). Predisposing factors for musculoskeletal symptoms in intensive care unit nurses. Int. Nurs. Rev..

[27] Alexopoulos E.C., Burdorf A., Kalokerinou A. (2003). Risk factors for musculoskeletal disorders among nursing personnel in Greek hospitals. Int. Arch. Occup. Environ. Health.

[28] Alexopoulos E.C., Burdorf A., Kalokerinou A. (2006). A comparative analysis on musculoskeletal disorders between Greek and Dutch nursing personnel. Int. Arch. Occup. Environ. Health.

[29] Arvidsson I., Gremark Simonsen J., Dahlqvist C., Axmon A., Karlson B., Bjork J., Nordander C. (2016). Cross-sectional associations between occupational factors and musculoskeletal pain in women teachers, nurses and sonographers. BMC Musculoskelet. Disord..

[30] Freimann T., Coggon D., Merisalu E., Animagi L., Paasuke M. (2013). Risk factors for musculoskeletal pain amongst nurses in Estonia: A cross-sectional study. BMC Musculoskelet. Disord..

[31] Nützi M., Koch P., Baur H., Elfering A. (2015). Work-Family Conflict, Task Interruptions, and Influence at Work Predict Musculoskeletal Pain in Operating Room Nurses. Saf. Health Work..

[32] Passali C., Maniopoulou D., Apostolakis I., Varlamis I. (2018). Work-related musculoskeletal disorders among Greek hospital nursing professionals: A cross-sectional observational study. Work.

[33] Ribeiro T., Serranheira F., Loureiro H. (2017). Work related musculoskeletal disorders in primary health care nurses. Appl. Nurs. Res..

[34] Serranheira F., Sousa-Uva M., Sousa-Uva A. (2015). Hospital nurses tasks and work-related musculoskeletal disorders symptoms: A detailed analysis. Work.

[35] Westergren E., Ludvigsen M.S., Lindberg M. (2021). Prevalence of musculoskeletal complaints among haemodialysis nurses—A comparison between Danish and Swedish samples. Int. J. Occup. Saf. Erg..

[36] Mailutha J., Mugga J., Kanali C.L. (2020). Prevalence of Musculoskeletal Disorders among Nurses in Kenya: Part 1, Anthropometric Data and MSDS. Int. J. Emerg. Technol. Adv. Eng..

[37] Al-samawi M.A.G., Ahmed H.M., Awad A.A. (2015). Incidences of Low Back Pain among Nurses Working in Elmak Nimer University Hospital—Shendi—Sudan 2015. Nurs. Health.

[38] Bernal D., Campos-Serna J., Tobias A., Vargas-Prada S., Benavides F.G., Serra C. (2015). Work-related psychosocial risk factors and musculoskeletal disorders in hospital nurses and nursing aides: A systematic review and meta-analysis. Int. J. Nurs. Stud..

[39] Gorce P., Jacquier-Bret J. (2024). Work-Related Musculoskeletal Disorders Prevalence among American, Asian and European Surgeons during Robotic/Video-Assisted Surgery. Int. J. Phys. Med. Rehabil..

[40] Berguer R., Rab G.T., Abu-Ghaida H., Alarcon A., Chung J. (1997). A comparison of surgeons’ posture during laparoscopic and open surgical procedures. Surg. Endosc..

[41] Droeze E., Jonsson H. (2005). Evaluation of ergonomic interventions to reduce musculoskeletal disorders of dentists in the Netherlands. Work.

[42] Alexopoulos E., Stathi I.-C., Charizani F. (2004). Prevalence of musculoskeletal disorders in dentists. BMC Musculoskelet. Disord..

[43] Gorce P., Jacquier-Bret J. (2023). Effect of Assisted Surgery on Work-Related Musculoskeletal Disorder Prevalence by Body Area among Surgeons: Systematic Review and Meta-Analysis. Int. J. Environ. Res. Public Health.

[44] Vieira E.R., Schneider P., Guidera C., Gadotti I.C., Brunt D. (2016). Work-related musculoskeletal disorders among physical therapists: A systematic review. J. Back. Musculoskelet. Rehabil..

[45] Gorce P., Jacquier-Bret J. (2024). A systematic review of work related musculoskeletal disorders among physical therapists and physiotherapists. J. Bodyw. Mov. Ther..

[46] Almeida M.B., Povoa R., Tavares D., Alves P.M., Oliveira R. (2023). Prevalence of musculoskeletal disorders among dental students: A systematic review and meta-analysis. Heliyon.

[47] Chenna D., Pentapati K.C., Kumar M., Madi M., Siddiq H. (2022). Prevalence of musculoskeletal disorders among dental healthcare providers: A systematic review and meta-analysis. F1000Research.

[48] Cao W., Hu L., He Y., Yang P., Li X., Cao S. (2021). Work-Related Musculoskeletal Disorders Among Hospital Midwives in Chenzhou, Hunan Province, China and Associations with Job Stress and Working Conditions. Risk Manag. Healhc. Policy.

[49] Kuorinka I., Jonsson B., Kilbom A., Vinterberg H., Biering-Sørensen F., Andersson G., Jørgensen K. (1987). Standardised Nordic questionnaires for the analysis of musculoskeletal symptoms. Appl Erg..

